# A Case Series of Hemophagocytic Lymphohistiocytosis: An Atypical Presentation of Visceral Leishmaniasis

**DOI:** 10.7759/cureus.58237

**Published:** 2024-04-14

**Authors:** Mark Grigoryan, Violeta Manukyan, Saten Hovhannisyan, Hripsime Apresyan

**Affiliations:** 1 Infectious Diseases, Muratsan University Hospital Complex, Yerevan, ARM; 2 Infectious Diseases, Yerevan State Medical University, Yerevan, ARM; 3 Pediatric Oncology, Yeolyan Hematology and Oncology Center, Yerevan, ARM

**Keywords:** high dose of liposomal amphotericin b, hscore, visceral leishmaniasis, hlh, hemophagocytic lymphohistiocytosis

## Abstract

Visceral leishmaniasis (VL) is a parasitic vector-borne disease endemic in Armenia. Its complications include hemophagocytic lymphohistiocytosis (HLH), which is a potentially fatal syndrome if misdiagnosed or left untreated. Higher clinical caution is required for the prompt diagnosis of HLH since the clinical findings associated with systemic inflammation overlap with those of many other pathological conditions, such as sepsis or Kawasaki disease. This study aims to provide an overview of the most common presentations that should prompt consideration of HLH. We described a case series of three pediatric patients with VL who developed HLH during antiparasitic treatment and received total doses of 40 mg/kg of liposomal amphotericin B for complete elimination of the pathogen.

## Introduction

Visceral leishmaniasis (VL) is a protozoal vector-borne disease that affects both humans and animals. The pathogen is an intracellular parasite of the genus *Leishmania *and is transmitted by certain species of sandflies, namely *Phlebotomus *and *Lutzomyia *[1,2]. Dogs are the main reservoir in Armenia and are responsible for maintaining and circulating these species. VL is the second most lethal neglected tropical and subtropical disease [3]. Annually 500,000 newly diagnosed cases of VL are reported worldwide [4].

The incidence rate of VL in Armenia is 0.03-0.6 per 100,000 people, the highest among children up to 3 years old [5]. The signs and symptoms of VL are fever, weight loss, failure to thrive, and hepatosplenomegaly. Patients may present with hematological abnormalities like anemia, leukopenia, and thrombocytopenia [6]. Patients with VL are prone to secondary infections due to several abnormalities associated with VL, including neutropenia, septicemia, uncontrolled bleeding due to thrombocytopenia and coagulopathy, and splenic rupture. Late-stage complications include peripheral edema and cachexia. VL infrequently can be complicated by a complex syndrome of hemophagocytic lymphohistiocytosis (HLH).

HLH is a life-threatening syndrome caused by either excessive macrophage-monocyte-histiocyte system activation or the absence of normal downregulation of cytokine-releasing immune cells. HLH occurring in patients with identified gene mutations linked to granule-dependent cytotoxicity are referred to as "primary" or familial HLH (FHL) [7]. The International Union of Immunological Societies (IUIS) defines FHL as inborn errors of immunity (IEI) primarily characterized by HLH. On the other hand, if HLH is provoked by infections, autoimmune manifestations, or malignancy without the presence of particular mutations in genes associated with FHL, it is labeled as "secondary" or "acquired." Nevertheless, this classification might be seen as too unsophisticated, as infections or other immune-stimulating factors frequently initiate primary HLH, while secondary HLH could obscure undetected or unidentified genetic factors. Furthermore, since both primary and secondary HLH arise from an imbalance in immune regulation, various IEIs besides FHL could contribute to the predisposition to HLH [8]. However, the terms "primary HLH" and "secondary HLH" have led to considerable confusion since infections or other immune-activating events can provoke both primary and secondary HLH and gene mutations can be identified among individuals of any age and with any family history. North American Consortium for Histiocytosis (NACHO) recommends the following terminology: HLH syndrome is characterized by abnormal immune activation, frequently linked with genetic abnormalities affecting lymphocyte cytotoxicity; HLH disease refers to the manifestation of HLH syndrome where the primary issue lies in the distinctive immune activation and can be linked to specific genetic and/or environmental factors [9]. The most extensive data on primary HLH originates from a Swedish national registry, which gathered information from 1987 to 2006. This registry indicated an annual incidence of around 1.5 per million [10]. The prevalence is higher in 0-18 months of age. The clinical manifestation of HLH may resemble symptoms seen in infections, unexplained fever, hepatitis, or encephalitis [11]. It is essential to include VL in the list of differential diagnoses since VL is among the infectious causes of HLH and becomes particularly important in endemic regions [12]. It is challenging to diagnose HLH in a patient with VL whose condition is worsening despite ongoing treatment. Diagnosis of HLH may be missed or delayed, as fever, hematological abnormalities, and hepatosplenomegaly can be attributed to leishmaniasis itself or even Amphotericin B-causing febrile infusion reactions and cytopenias.

We described a case series of three pediatric patients with VL who developed secondary HLH during antiparasitic treatment. To diagnose HLH, we used an HScore calculator created by Dr. Laurence Fardet and/or HLH-2004 protocol diagnostic criteria [13]. Two patients received corticosteroids in addition to liposomal amphotericin B (LAMB). To get rid of the primary disease (i.e., infection) we had to use a higher dose of LAMB as indicated in patients with human immunodeficiency virus coinfection [14].

## Case presentation

Case 1

In May 2021, a 22-month-old male from the Russian Federation was hospitalized at the Muratsan University Hospital due to a fever lasting seven days, peaking at 40°C, weakness persisting for five months, and abdominal distention. Over the past two days, the patient experienced three to four episodes of vomiting without bile. The physical examination revealed an ill-appearing child with skin pallor. The liver was palpable 3 cm below the costal margin and the spleen was palpable in the left lower quadrant of the abdomen.

**Figure 1:**
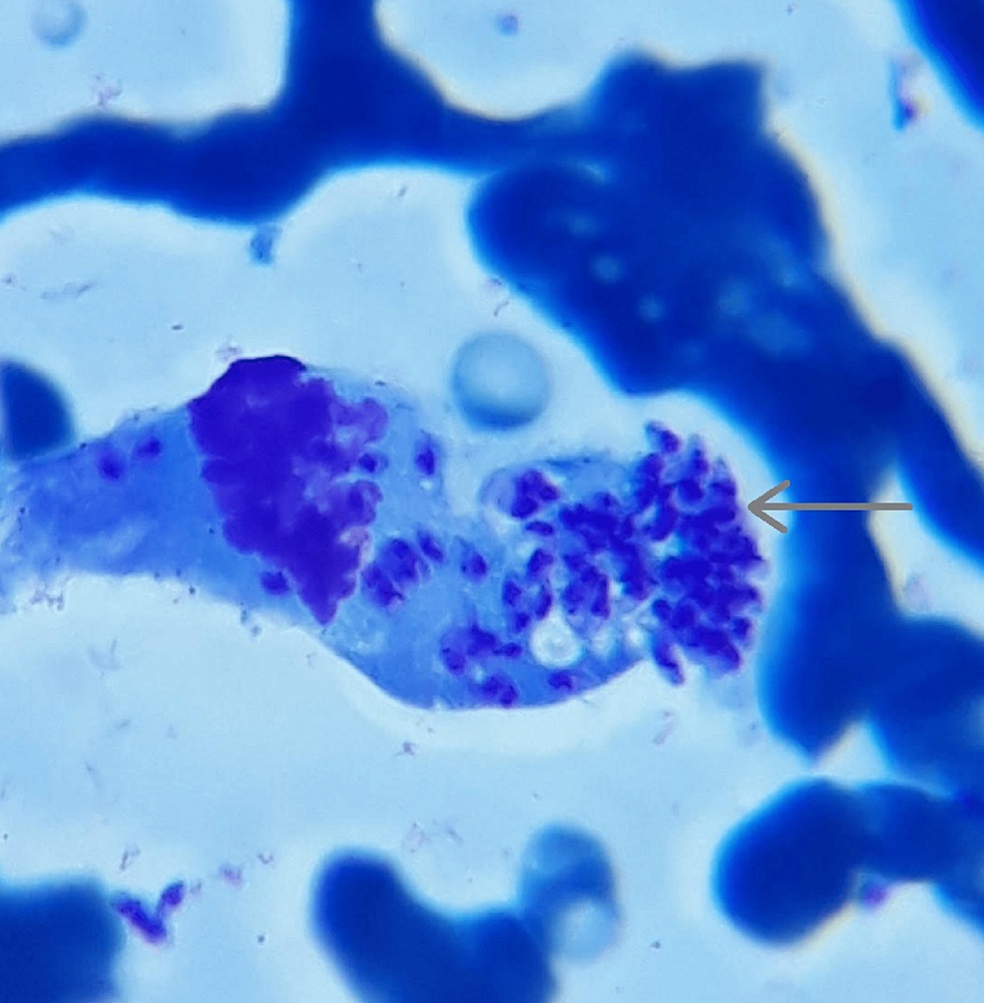
Leishmania organisms (Giemsa stain) on microscopic examination (100x magnification)

The patient was diagnosed with VL five months ago and responded well to the treatment with a seven-day course of LAMB (21 mg/kg) in Moscow. However, four months following the completion of therapy, the patient experienced symptoms, prompting the family to move to Armenia for further diagnosis and treatment. His traveling history included a trip to Makhachkala, Republic of Dagestan, where Leishmaniasis is endemic, suggesting that symptomatic travelers from this region should undergo testing for VL [15]. Therefore, suspecting a recurrence of VL, a bone marrow biopsy was conducted, revealing the presence of *Leishmania *organisms upon microscopic inspection (Figure 1). Additionally, the *Leishmania *IgM/IgG rapid test yielded positive results.

On the third day of admission, a complete blood count (CBC) showed anemia (8.4 g/dL), neutropenia (1.18x10^9^/L) and thrombocytopenia (86x10^9^/L). White blood cell count was 11.73x10^9^/L (N=4.0-10.0x10^9^/L) with lymphocytosis 7.59x10^9^/L (N=1.0-3.0x10^9^/L), and monocytosis 2.95x10^9^/L (N=0.2-0.8x10^9^/L). No atypical lymphocytes were detected. Peripheral blood smear showed platelets of 104x10^9^/L (35‰). According to the blood workup mean corpuscular volume was 76.4 fL (N=80-100 fL), reticulocyte count - 3.0% (N=0.5-2.5%), serum iron - 9.4 µmol/L (N=10.5-30.4 µmol/L), lactate dehydrogenase - 506 U/L (N=120-320 U/L). The laboratory data exposes that the mechanism of anemia is predominantly hemolytic. However, the reduced plasma iron level in the presence of increased iron stores suggests abnormal iron retention by macrophages, characteristic of anemia of chronic disease. The metabolic panel included hypertriglyceridemia (3.21, N=0.45-1.8 mmol/L), hyperferritinemia (2563.2, N=14-124 µg/L), and transaminitis (more than twice the normal range) (Table 1).

**Table 1:** Laboratory data on the third day of admission (case 1)

Test	On third day of admission	Reference	Unit
White blood cells (WBC)	11.73	4.0-10.0	x10^9^/L
Neutrophils	1.18	1.8-6.4	x10^9^/L
Lymphocytes	7.59	2.0-4.0	x10^9^/L
Monocytes	2.95	0.2-0.8	x10^9^/L
Hemoglobin (HGB)	8.4	11.0-16.0	g/dL
Platelets (PLT)	86	100-300	x10^9^/L
Reticulocytes	3.0	0.5-2.5	%
Serum iron	9.4	10.5-30.4	µmol/L
Ferritin	2563.2	14-124	ng/mL
Lactate dehydrogenase (LDH)	506	120-320	U/L
Alanine aminotransferase (ALT)	81.4	<40	U/L
Aspartate aminotransferase (AST)	180.1	<35	U/L
Triglycerides	3.21	0.45-1.8	mmol/L

**Figure 2:**
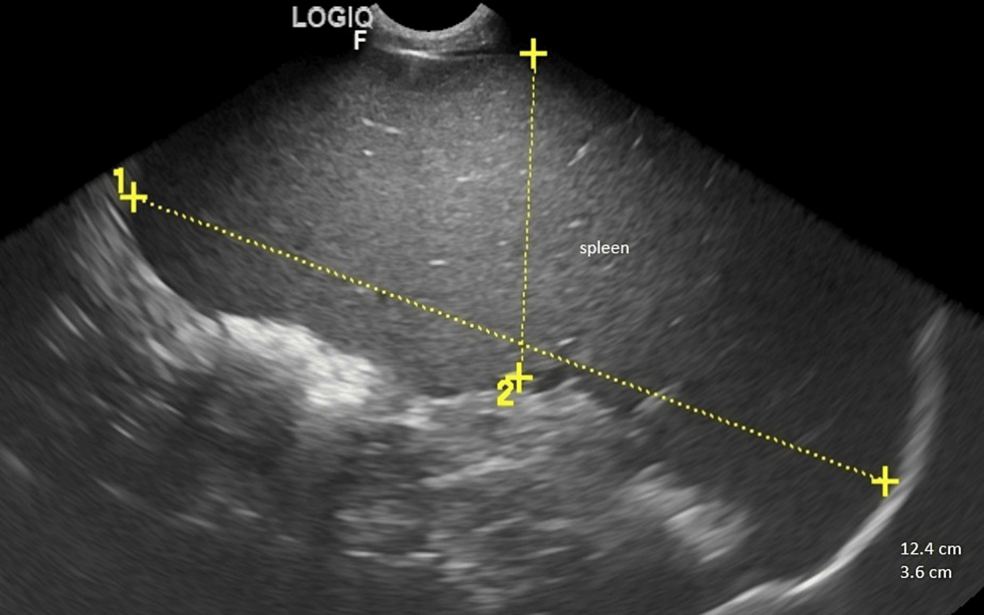
Ultrasonography revealed massive splenomegaly (12.4x3.6 cm, N<5x3 cm)

Ultrasonography revealed massive splenomegaly (12.4x3.6 cm, N<5x3 cm) and hepatomegaly (Figure 2).

Blood and urine cultures were obtained and empiric treatment with intravenous Amoxicillin-Clavulanate was initiated. The patient's EBV VCA IgM was negative. He had no conjunctivitis, rash, lymphadenopathy, or tonsilitis which made Kawasaki disease or infectious mononucleosis unlikely. Considering the diagnosis of HLH bone marrow aspiration was performed; however, hemophagocytes were not detected. Both blood and urine cultures were negative. Calculating the HScore, the probability of HLH was 88-93% (209 points). In immunocompetent patients with VL who do not show improvement after initial treatment using LAMB, alternative drugs, higher doses or extended courses of LAMB, or combination regimens should be considered for treatment [16,17]. The patient received LAMB of 40 mg/kg total dose during 18 days. He was afebrile since the fifth day of admission and was discharged from the hospital after completing the treatment.

Case 2

A 13-month-old male was presented to the emergency department from Tavush province (an endemic region of VL in Armenia). The patient complained of a five-day fever of 40°C, loss of appetite, vomiting after meals, and diarrhea episodes up to five times daily. Physical examination showed skin pallor and peripheral edema. The abdominal examination revealed the liver palpable 4 cm below the costal margin and the tip of the spleen palpable in the left lower quadrant of the abdomen. A CBC revealed pancytopenia (hemoglobin - 4.6 g/dL, WBC - 2.38x10^9^/L, neutrophils - 0.27x10^9^/L, platelets - 12x10^9^/L), the liver function tests were remarkable with aspartate aminotransferase (AST) of 350 U/L and alanine aminotransferase (ALT) of 250 U/L. Ferritin level was more than 2000 µg/L (N=13-350). Prothrombin time was 47.4 seconds (N=12-16) and the international normalized ratio (INR) was elevated to 5.4 (N=1.0-2.0) (Table 2).

**Table 2:** Laboratory data (case 2) aPTT: Activated partial thromboplastin time; PT: Prothrombin time; INR: International normalized ratio

Test	At the time of admission	On the 3rd day of admission	After a 10-day course of Liposomal Amphotericin B	On the 40th day of admission	Reference	Unit
Neutrophil	0.27	0.18	1.01	0.34	1.8-6.4	x10^9^/L
Hemoglobin	4.6	4.5	7.34	7.7	11.0-16.0	g/dL
Platelets	12	15	191	94	100-300	x10^9^/L
Ferritin	>2000	>2000	-	1070	14-124	ng/mL
Alanine aminotransferase (ALT)	250	17.1	-	22.7	<40	U/L
Aspartate aminotransferase (AST)	350	107.1	-	50.9	<35	U/L
aPTT	>120	68.6	62.8	67.3	28-44.5	Second
PT	47.4	26.2	11.8	16.8	11-14.5	Second
INR	5.4	2.61	1.34	1.92	1.0-2.0	
Fibrinogen	-	-	2.71	0.86	2.0-4	g/L
Triglycerides	-	-	-	5.4	0.45-1.8	mmol/L

**Figure 3:**
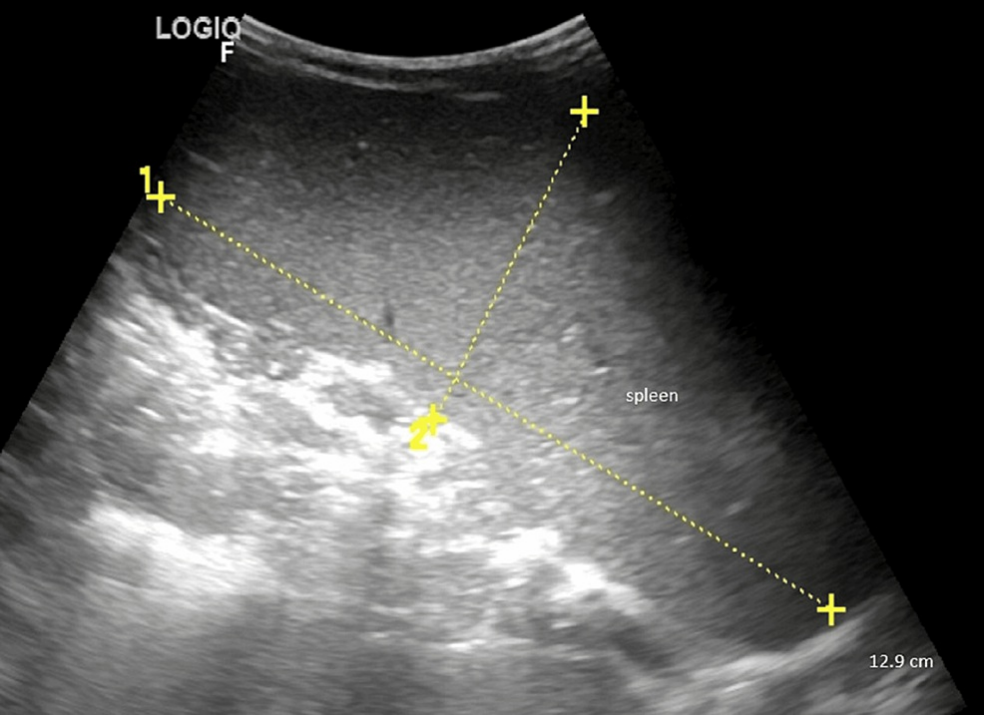
Ultrasonography of the abdomen showed hepatomegaly and splenomegaly of 12.9 cm (N<7)

Ultrasonography of the abdomen showed hepatomegaly (maximum dimension was 9.15 cm, N<8.5) and splenomegaly of 12.9 cm (N<7) (Figure 3).

Bone marrow aspirate (BMA) examination revealed Leishman-Donovan (LD) bodies but did not show hemophagocytes. LAMB was commenced. A total of 21 mg/kg of LAMB was given during 10 days. Clinical and laboratory (hemoglobin (HGB)- 7.34 g/dL, neutrophil - 1.01x10^9^/L, platelets - 191x10^9^/L) improvement was seen. Peripheral blood smear showed anisocytosis and macrocytosis, and LDH was 368 U/L. The spleen size was reduced to 9.67 cm. On the 40th day of hospitalization, the patient spiked a fever of 40°C, and his condition deteriorated. CBC and comprehensive metabolic panel (CMP) were performed. CBC indicated anemia (HGB - 7.7 g/dL, RBC - 2.39x10^9^/L), neutropenia (0.34x10^9^/L), and thrombocytopenia (94x10^9^/L). CMP revealed hyperferritinemia (1070 µg/L, N=13-350), and hypertriglyceridemia (5.18 mmol/L, N=0.45-1.8). AST level was elevated (50.9U/L). Activated partial thromboplastin time, prothrombin time, INR, and fibrinogen levels were 67.3 seconds (N=26-36), 16.8 seconds (N=11-14.5), 1.92 (N=1.0-2.0) and 0.86 g/L (N=2-4), respectively (Table 2). Ultrasonography showed splenohepatomegaly (liver - 9.29 cm, spleen - 13.17 cm). Blood and urine cultures were drowned. Ceftriaxone was started empirically. No hemophagocytes were seen on BMA smears. Both blood and urine cultures provided negative results, indicating no microbial growth. HScore was calculated as 234, and five (fever, splenomegaly, cytopenias, hyperferritinemia >500 µg/L, hypertriglyceridemia >3 mmol/L, and/or hypofibrinogenemia) out of eight criteria of the HLH 2004 protocol were met. Considering the immunosuppression and possible treatment failure the patient received a total of 40 mg/kg of LAMB. Dexamethasone of 0.2 mg/kg/day was given initially for 12 days, followed by 0.1 mg/kg/day for seven days. IVIG was given 400 mg/kg/dose and administered for a day. On day 11 of the immunosuppression, the CMP was normal and the patient became afebrile on the 14th day of fever onset. He was discharged with clinical improvement.

Case 3

A previously healthy six-year-old boy was admitted to the hospital with a four-day fever of 39.5°C, asthenia, and pain in the right upper quadrant of the abdomen which had started eight days ago. Physical examination revealed abdominal protuberance, the liver was palpated 3 cm below, and the spleen 5 cm below the costal margin. Hematologic examinations showed pancytopenia, elevated C-reactive protein (94.9 mg/L, normal range<5), and transaminitis (AST - 113.0 U/L, ALT - 46.8 U/L). The coagulation profile was within normal ranges. Abdominal ultrasonography revealed hepatosplenomegaly. The patient was checked for the VL rk39 and antileishmanial IgM/IgG rapid tests which both were positive. Despite the antiparasitic treatment (LAMB), on the fifth day of admission, the patient’s clinical conditions and laboratory values deteriorated, with an elevated frequency of fever spikes. Blood and urine cultures were pending. We compared CBC results on the third and fifth days of admission: HGB of 8.3 vs. 6.2 g/dL, platelets of 81x10^9^ vs. 60x10^9^/L, neutrophil 1.43x10^9^ vs. 0.60x10^9^/L. CMP showed hyperferritinemia of higher than 3000 ng/mL and hypertriglyceridemia of 6.3 mmol/L. Both procalcitonin (1.6 ng/mL, N<0.05) and interleukin-6 (89.2 pg/mL, N<7.0) levels were elevated (Table 3). Blood and urine cultures were negative.

**Table 3:** Laboratory data (case 3)

Test	At the time of admission	On the third day of admission	On the fifth day of admission	Reference	Unit
Neutrophil	1.1	1.43	0.60	1.8-6.4	x10^9^/L
Hemoglobin	7.4	8.3	6.2	11.0-16.0	g/dL
Platelets	92	81	60	100-300	x10^9^/L
C-reactive protein (CRP)	94.9	-	-	0-5	mg/L
Ferritin	-	-	>3000	14-124	ng/mL
Alanine aminotransferase (ALT)	46.8	-	-	<40	U/L
Aspartate aminotransferase (AST)	113.0	-	-	<35	U/L
Triglycerides	-	-	6.3	0.45-1.8	mmol/L
Procalcitonin (PCT)	-	-	1.6	0-0.05	ng/mL
Interleukin-6 (IL-6)	-	-	89.2	<7	pg/mL

HLH was diagnosed as five out of eight HLH-2004 protocol criteria met, and HScore was 239. Dexamethasone at a dosage of 0.2 mg/kg/day was initiated. However, there was no improvement observed after 48 hours, leading to a switch to intermittent administration of Methylprednisolone at a dosage of 500 mg every other day for three days. This was followed by continuous administration of Methylprednisolone at a dosage of 1 mg/kg for five days. Corticosteroid therapy was discontinued after a tapering period of six days. Treatment with LAMB was continued to a total of 38 mg/kg [14,16,17]. On the 12th day of admission, the boy was afebrile and clinically stable. After 20 days of hospitalization, the patient was discharged to home.

## Discussion

VL is a potentially fatal vector-borne protozoal infection affecting a variety of internal organs [18]. In 2005, VL treatment with LAMB of a total of 21 mg/kg was considered adequate in immunocompetent patients [19]. Immunocompetent patients not responding to initial therapy with LAMB should be treated with an alternative drug, a higher dose or longer LAMB course, or a combination regimen [16,17].

Rarely VL can cause secondary HLH which is a life-threatening condition requiring early recognition and treatment [20]. Higher clinical suspicion is necessary for the prompt diagnosis of HLH because the clinical findings associated with systemic inflammation overlap with those of VL. Careful consideration of clinical presentation, laboratory findings, diagnostic tests, response to treatment, and clinical course can help differentiate the severity of VL from sepsis and HLH. Secondary causes of HLH include infections, malignancies, autoimmune conditions, and immune deficiencies. Any infection virtually can be associated with HLH; however, DNA-stranded viruses and intracellular pathogens (e.g., Leishmania) are the most commonly reported. It is considered that HLH develops due to abnormal reciprocal activation of mononuclear phagocytes (MNPs) (monocytes, macrophages, and dendritic cells) and type 1 lymphocytes (NK cells and Th1, CD8, and NKT cells) [20].

For more than a decade, physicians all over the world have used HLH-2004 protocol diagnostic criteria. HLH diagnosis is made when (A) a genetic defect consistent with HLH is detected or (B) five out of eight clinical and laboratory criteria fulfilled: fever, splenomegaly, cytopenia ≥2 cell lineages, hypertriglyceridemia or hypofibrinogenemia, hyperferritinemia, soluble CD25 >2400 U/mL, hemophagocytosis in bone marrow, spleen, lymph nodes, or liver, and low or absent NK-cell cytotoxicity (Table 4).

**Table 4:** Diagnostic criteria guideline of the HLH-2004 protocol HLH: Hemophagocytic lymphohistiocytosis; NK-cell: Natural killer cell

(A) Genetic defect consistent with HLH or (B) Five out of eight clinical and laboratory criteria fulfilled
Fever
Splenomegaly
Cytopenia ≥2 cell lineages
Hemoglobin <9.0 g/dL, in neonates <10.0 g/dL
Platelet count <100×10^9^/L
Neutrophil count <1×10^9^/L
Hypertriglyceridemia (>3 mmol/L) or hypofibrinogenemia (<1.5 g/L)
Hyperferritinemia (>500 µg/L)
Soluble CD25 >2400 U/mL (or elevated compared with laboratory-defined normal ranges)
Hemophagocytosis in bone marrow, spleen, lymph nodes, or liver
Low or absent NK-cell cytotoxicity

An alternative scoring system has been developed to generate a diagnostic score referred to as an "Hscore" that estimates the probability of HLH. This incorporates points for immunosuppression, fever, organomegaly, levels of triglycerides, ferritin, ALT, and fibrinogen, degree of cytopenias, and presence of hemophagocytosis on the BMA (Table 5) [13].

**Table 5:** Criteria for HScore calculation developed by Dr. Fardet HIV: Human immunodeficiency virus; SGOT: Serum glutamic-oxaloacetic transaminase; AST: Aspartate aminotransferase

N	Criteria	Options	Score
1	Known underlying immunodepression (HIV positive or receiving long‐term immunosuppressive therapy (i.e., glucocorticoids, cyclosporine, azathioprine))	Yes/no	+18/0
2	Maximal temperature (°C)	Strictly <38.4/38.4-39.4/>39.4	0/+33/+49
3	Organomegaly	No/hepatomegaly or splenomegaly/hepatosplenomegaly	0/+23/+38
4	Lower hemoglobin level	≤9.2 g/dL/>9.2 g/dL	one lineage- 0/two lineages +24/three lineages +34
5	Lower leukocytes count	≤5000/mm^3^/>5000/mm^3^	
6	Lower platelets count	≤100000/mm^3^/>100000/mm^3^	
7	Higher ferritin level (ng/mL)	<2000/2000-6000/>6000	0/+35/+50
8	Higher triglyceride level mg/dL (mmol/L)	<132.7 (<1.5)/132.7-354 (1.5-4)/>354 (>4)	0/+44/+64
9	Lower fibrinogen level (g/L)	>2.5/≤2.5	0/+30
10	Higher SGOT/AST level (UI/L)	<30/≥30	0/+19
11	Hemophagocytosis features on bone marrow aspirate	No/yes	0/+35

Rajagopala et al found that another confounding element is the negativity of the first bone marrow aspiration in 64.7% of HLH and 36.3% of LD bodies [11]. The low sensitivity and specificity of the existence of hemophagocytosis make it a non-obligatory criterion for diagnosing HLH [21]. An HScore equal to or greater than 250 indicates a 99% likelihood of having HLH, while a score of 90 or lower indicates a less than 1% chance of having HLH [22].

All three patients follow-up examinations after 1, 3, 6, 12, and 24 months of discharge from the hospital were unremarkable. We routinely performed physical examinations, reviewed CBC and liver function test results, evaluated any reported symptoms, provided education on lifestyle modifications, and maintained open communication with the patient’s general pediatrician for coordinated care.

## Conclusions

Features of HLH should be considered in all serious infections, and infectious disease consultation may be appropriate in most HLH patients. HLH is an under-recognized disease due to its clinical course similarity with infectious diseases, malignancies, toxic shock syndrome, and Kawasaki disease. HLH-2004 criteria and the HScore calculator by Dr. Fardet are helpful tools for HLH suspicion. Early recognition and treatment of the disease can lead to improved patient outcomes. Continuation of LAMB treatment beyond the standard total dose of 21 mg/kg is warranted in cases of disease relapse, treatment failure associated with HLH, and immunosuppression. Considering the immune homeostasis alterations in HLH, we propose treating VL with a dosage of 40 mg/kg of LAMB to reduce the risk of VL relapse.
